# Molecular Docking and Simulation-Based Exploration of Niclosamide as a Potential Inhibitor of the p62 ZZ Domain

**DOI:** 10.3390/biology15151290

**Published:** 2026-08-04

**Authors:** Yuki Hatayama, Hisashi Shimohiro, Koji Kawamura

**Affiliations:** 1 Division of Clinical Laboratory, Tottori University Hospital, Tottori 683-8504, Japan; 2 Department of Pathobiological Science and Technology, School of Health Science, Faculty of Medicine, Tottori University, Tottori 683-8504, Japan; 3 Division of Clinical Laboratory Medicine, Department of Multidisciplinary Internal Medicine, Faculty of Medicine, Tottori University, Tottori 683-8503, Japan

**Keywords:** acute myeloid leukemia, VTRNA1-1, niclosamide, drug repositioning, machine learning, explainable AI, p62, SQSTM1, MYC, transcriptomics

## Abstract

Acute myeloid leukemia (AML) remains a therapeutic challenge due to complex oncogenic networks, including the often-undruggable MYC pathway. Here, we present an integrated in silico framework combining transcriptomic analysis, machine learning, and molecular dynamics (MD) simulations to explore potential therapeutic approaches targeting vault RNA1-1 (VTRNA1-1) in AML. RNA-seq profiling revealed that VTRNA1-1 depletion is associated with a profound disruption of the MYC and FOXM1 regulatory axes. To highlight compounds capable of recapitulating this transcriptomic signature, we developed a machine learning pipeline utilizing a Random Forest classifier trained on a fully compiled L1000FWD database subset. Virtual screening of approved drugs predicted the anthelmintic niclosamide as a top candidate (98.17% mimic probability). Explainable AI further rationalized this prediction by highlighting specific fragments within niclosamide’s salicylanilide core. Furthermore, a 200 ns MD simulation indicated favorable computational stability of niclosamide bound to the p62 (SQSTM1) ZZ domain. The complex showed rapid structural convergence (ligand RMSD plateauing at 1.65 nm) without dissociation, while maintaining strict receptor compactness (steady Radius of Gyration and solvent-accessible surface area) and a persistent interaction network of ~73 close atomic contacts. These findings suggest that niclosamide may function as a stable physical “lid” over the p62 ZZ domain, occluding its N-degron-binding cleft. Taken together, our computational framework highlights niclosamide as a promising candidate for AML drug repurposing, providing a hypothesis-generating foundation that warrants rigorous experimental validation.

## Simple Summary

Acute myeloid leukemia (AML) is an aggressive blood cancer that urgently requires novel therapeutic strategies. In this study, we utilized computational modeling and docking simulations to investigate whether the pre-existing drug niclosamide could potentially target the p62 ZZ domain—a key protein involved in cancer cell survival—by mimicking the non-coding RNA vtRNA1-1. Our in silico findings predict that niclosamide may disrupt this critical survival pathway, establishing a promising computational framework to guide future experimental validation for AML treatments.

## 1. Introduction

Acute myeloid leukemia (AML) is a highly heterogeneous and aggressive hematological malignancy characterized by rapid proliferation of poorly differentiated myeloid precursors. Despite recent advances in targeted therapies, a significant proportion of patients experience relapse, highlighting the urgent need to identify novel therapeutic targets [1,2,3,4,5]. Recently, non-coding RNAs (ncRNAs) have emerged as critical regulators of oncogenesis. Among them, vault RNAs (VTRNAs), a class of small ncRNAs transcribed by RNA polymerase III, have gained attention because of their roles in multidrug resistance, apoptosis regulation, and cancer cell survival [6,7,8,9,10,11,12,13,14,15].

Our previous clinical investigations have established a strong association between vault RNA1-1 (VTRNA1-1) and hematological malignancies. We previously reported that serum VTRNA1-1 levels are significantly altered in patients with these disorders [16] and serve as reliable biomarkers of bone marrow cell density [17]. These clinical observations strongly suggest that VTRNA1-1 is not merely a passive byproduct shed by leukemic cells but is potentially an active driver of the disease. However, the precise intracellular function and therapeutic vulnerability of VTRNA1-1 in AML pathogenesis remain unclear.

To bridge the gap between clinical biomarker discovery and intracellular molecular mechanisms, we first investigated the biological role of VTRNA1-1 using transcriptomic data analysis. By analyzing global transcriptomic profiling (RNA-seq) data derived from VTRNA1-1 depletion models, we mapped extensive cellular dependencies on VTRNA1-1. We hypothesized that understanding the gene expression signature resulting from VTRNA1-1 depletion would provide a computational blueprint for novel therapeutic interventions.

To translate these transcriptomic findings into viable therapeutic strategies, we employed a data-driven drug repositioning approach. Using the Library of Integrated Network-based Cellular Signatures (LINCS) L1000FWD database [18], we developed a machine learning pipeline utilizing a Random Forest classifier to query our RNA-seq differential expression signature and identify FDA-approved compounds capable of mimicking the exact transcriptomic state of VTRNA1-1 depletion. This transcriptomics-guided screening aimed to identify a drug that could artificially induce the collapse of oncogenic signaling networks, including the MYC and FOXM1 regulatory axes, as observed in our transcriptomic models.

Biologically, VTRNA1-1 exerts its cellular functions by directly interacting with scaffold protein p62 (SQSTM1) [19,20,21,22,23]. Therefore, we hypothesized that any compound capable of phenocopying VTRNA1-1 depletion might do so by physically interfering with p62-mediated signaling hubs. In this study, we successfully identified the anthelmintic drug niclosamide using machine learning and the L1000FWD pipeline. To validate the structural and biophysical rationale of this prediction, we integrated Explainable AI (XAI) analysis with molecular docking and extensive 200 ns molecular dynamics (MD) simulations. We sought to determine whether niclosamide stably targets the conserved ZZ domain of p62 to physically occlude its N-degron-binding cleft. Ultimately, this study provides a logical and pure in silico framework bridging functional genomics, AI-driven drug repositioning, and structural bioinformatics and presents a novel computational rationale for a targeted therapeutic paradigm for AML.

## 2. Materials and Methods

### 2.1. Cell Culture and Lentiviral Knockdown

The human AML cell line, THP-1, was cultured in RPMI 1640 medium supplemented with 10% fetal bovine serum (FBS) and 1% penicillin/streptomycin. Cells were maintained in a humidified incubator at 37 °C with 5% CO_2_. The lentiviral vector for VTRNA1-1 knockdown was custom-designed and synthesized by VectorBuilder (Chicago, IL, USA; Vector ID: VB241201-1190rze) (Table S1). We utilized a mammalian miR30-shRNA knockdown lentiviral vector (pLV [miR30]) under the control of the CMV promoter. To allow for the visualization and identification of transduced cells, the vector featured a nuclear localization signal-tagged enhanced green fluorescent protein (NLS-EGFP) reporter. Cells were transduced at a multiplicity of infection of 10 in the presence of a Lenti-X Transduction Sponge (Takara Bio, Shiga, Japan) to enhance viral entry. Following 24 h of incubation, the transduction medium was replaced with fresh complete growth medium. The specific target sequence for hVTRNA1-1 within the miR30-shRNA scaffold was 5’-GTTACTTCGACAGTTCTTTAAT-3’. A scrambled sequence was employed as a negative control (sh-NC).

### 2.2. RNA Extraction, qPCR Validation, and Transcriptomic Data Acquisition

To investigate global transcriptomic changes induced by VTRNA1-1 depletion in AML, total RNA was extracted from the lentiviral-transduced THP-1 cells (sh-NC and sh-VTRNA1-1, each performed in independent biological triplicates) using the miRNeasy Tissue/Cells Advanced Mini Kit (QIAGEN) according to the manufacturer’s protocol. The initial concentration and purity of the extracted RNA were evaluated using a NanoDrop spectrophotometer. To validate the knockdown efficiency of VTRNA1-1, quantitative real-time PCR (qPCR) was performed using the Fast Dx Real-Time PCR Instrument (Applied Biosystems). The relative expression levels of VTRNA1-1 were calculated, with ACTB (β-actin) serving as the endogenous normalization control (Table S1). Following the in-house RNA extraction and quality assessment, aliquots of the total RNA samples (three biological replicates per group) were submitted to Eurofins Genomics for library preparation and transcriptome sequencing on an Illumina NovaSeq platform. Both library construction and sequencing were executed by the service provider under strict quality control protocols. Raw and processed sequencing datasets generated from this study were deposited in the NCBI Gene Expression Omnibus (GEO) database under accession number GSE336862. The sequencing data were processed and analyzed using the automated BioJupies web server for alignment, transcript quantification, and differential expression analysis. Differentially expressed genes (DEGs) resulting from VTRNA1-1 depletion were defined using robust statistical thresholds of |log2 (Fold Change)| > 1.0 and an adjusted *p*-value < 0.05. Furthermore, programmatic pathway enrichment analyses, including Kyoto Encyclopedia of Genes and Genomes (KEGG) and Gene Ontology (GO) via integrated Enrichr modules, were performed to map primary oncogenic and regulatory cascades disrupted by VTRNA1-1 loss.

### 2.3. Programmatic L1000FWD Database Query

To identify small-molecule compounds that mimic the transcriptomic state of VTRNA1-1 depletion, we systematically queried the Library of Integrated Network-Based Cellular Signatures (LINCS) L1000FWD. Significantly upregulated and downregulated DEGs derived from our RNA-seq analysis were used as input signatures. To ensure reproducibility and robust data handling, a signature similarity search was executed programmatically using a custom Python-based (version 3.10) analytical pipeline. We looped the input signatures and utilized the L1000FWD API (analyze function) to compute connectivity scores against thousands of established drug-induced transcriptomic profiles. Comprehensive signature similarity search results (comprising 1461 valid compounds) were extracted as CSV files for quantitative ranking. These compounds, categorized by their connectivity scores, served as foundational training datasets for the subsequent machine learning models.

### 2.4. Machine Learning Model Construction, Virtual Screening, and XAI

To identify structurally diverse VTRNA1-1 mimics among approved drugs, we developed a machine-learning-based virtual screening pipeline.

Dataset Preparation and Feature Extraction: Based on the L1000FWD connectivity scores, the 1461 compounds were classified as “Mimic” (Class 1) or “Non-mimic” (Class 0/−1). The Simplified Molecular Input Line Entry System (SMILES) strings for all compounds were converted into Morgan fingerprints (2048 bits, radius = 2) using the open-source cheminformatics library, RDKit. This process encodes each molecular structure into 2048 binary features representing the presence or absence of specific chemical substructures.

A Random Forest classifier was trained on these features using the scikit-learn library in Python (version 3.10). To ensure absolute reproducibility of the model construction, the ensemble model was initialized with 100 estimators (n_estimators = 100) and trained using a fixed random seed (random_state = 42). To evaluate node splitting, the maximum depth parameter was specified without restriction (max_depth = None), and the default Gini impurity was utilized to measure the quality of splits. To strictly prevent data leakage and overfitting, model evaluation was conducted using an independent, unseen test dataset during cross-validation, ensuring complete separation between the feature extraction, training, and validation phases. When evaluated using an unseen test dataset during cross-validation, the model achieved an overall predictive accuracy of 71.67%. For the critical “Mimic” group (Class 1), the model achieved both a precision and recall of 0.69, validating its robust utility for initial virtual screening. Following this rigorous validation, the optimized Random Forest architecture was trained on the fully compiled 1461-compound dataset prior to execution against the ChEMBL database to maximize predictive power for production virtual screening.

Virtual Screening and Explainable AI (XAI): We retrieved a library of approved drugs (phase IV) from the ChEMBL database. The molecular fingerprints of these compounds were fed into our trained Random Forest model to predict the probability of each drug acting as a VTRNA1-1 mimic. This AI-driven screening identified the anthelmintic drug niclosamide as the top-ranked candidate. Furthermore, we employed the XAI approach to elucidate the structural rationale behind the predictions of AI. Feature importance scores derived from the Random Forest model were used to identify and visualize the 10 most influential molecular substructures (Morgan fingerprint bits) contributing to the “Mimic” classification.

### 2.5. Protein Preparation and In Silico Molecular Docking (PyRx)

Receptor Preparation: The 3D crystal structure of the human p62 (SQSTM1) ZZ domain was retrieved from the Protein Data Bank (PDB ID: 4MJM). To ensure optimal docking conditions, the structure was pre-processed using the PDB2PQR server (pH 7.0) to remove impurities such as water molecules and crystallization agents. Hydrogen atoms were added based on the AMBER force field, and the structure was converted to the pdbqt format.

Ligand Preparation: The 3D niclosamide conformer was obtained from PubChem (CID: 4477). Energy minimization was conducted using a Universal Force Field (UFF) via Open Babel within PyRx, followed by conversion to pdbqt format.

Docking Parameters: In silico molecular docking was performed using PyRx with an AutoDock Vina (version 1.1.2) engine. To allow an unbiased search for the optimal binding site, we conducted a blind docking simulation by setting a grid box encompassing the entire protein surface. Exhaustiveness was set to eight. The docking pose with the lowest binding energy (kcal/mol) and a root-mean-square deviation (RMSD) of zero was selected as the optimal conformation for downstream interaction analysis.

### 2.6. Interaction Visualization (Discovery Studio)

The molecular interactions of the optimal docking complex were visualized and analyzed using BIOVIA Discovery Studio Visualizer (v21.1). Both 3D spatial conformations and 2D interaction maps were generated to characterize the specific binding forces, including conventional hydrogen bonds, carbon–hydrogen bonds, and hydrophobic (pi–alkyl) interactions between niclosamide and the amino acid residues of the p62 ZZ domain.

### 2.7. Molecular Dynamics Simulations

Molecular dynamics (MD) simulations were performed using GROMACS (version 2026) to evaluate the structural stability and dynamic behavior of the p62 ZZ domain–niclosamide complex. The protein topology and coordinate files were generated using the AMBER force field, whereas the ligand parameters for niclosamide were generated with the General AMBER Force Field (GAFF) using ACPYPE. Partial atomic charges were assigned using the AM1-BCC method.

The protein–ligand complex was solvated in a cubic box with TIP3P water molecules, ensuring a minimum distance of 1.0 nm between the complex and the box edges. The system was neutralized by adding counterions to achieve a physiological salt concentration.

Energy minimization was performed using the steepest descent algorithm until the maximum force converged to less than 1000 kJ/mol/nm. The system was subsequently equilibrated under constant-number-of-particles, volume, and temperature (NVT) and constant-number-of-particles, pressure, and temperature (NPT) ensembles for 100 ps each. Temperature was maintained at 300 K using a modified Berendsen thermostat, and the pressure was regulated at 1.0 bar using the Parrinello–Rahman barostat.

Production MD simulations were performed for 200 ns with a 2 fs integration time. All covalent bonds involving hydrogen atoms were constrained using the LINCS algorithm. Short-range electrostatic and van der Waals interactions were calculated using a cutoff radius of 1.0 nm, whereas long-range electrostatic interactions were treated using the particle-mesh Ewald (PME) method. Periodic boundary conditions were applied in all three dimensions.

### 2.8. Trajectory and Post-MD Analysis

Prior to trajectory analysis, water molecules and ions were stripped from the trajectory using the *gmx trjconv* module to focus exclusively on the protein–ligand dynamics. Structural stability and conformational changes were monitored over a 200 ns timeline using GROMACS standard tools.

Root-mean-square deviation (RMSD) for both the protein backbone and the ligand and root-mean-square fluctuation (RMSF) for individual amino acid residues were calculated to determine structural convergence and local flexibility. The compactness and structural behavior of the complex were assessed based on the Radius of Gyration (Rg) and solvent-accessible surface area (SASA).

The persistence of the docking mode was verified by monitoring the number of close contacts between the protein and the ligand within a threshold of 0.35 nm using the *gmx mindist* module as a proxy for hydrogen bond occupancy. Additionally, temporal variations in protein secondary structures were evaluated using the integrated *gmx dssp* module inherent in GROMACS 2026. All the data profiles were plotted using the Metaplotlib library in Python (version 3.10).

### 2.9. Binding Free Energy Calculations

To quantify the thermodynamic driving forces and binding affinity of niclosamide to the target protein, Molecular Mechanics–Generalized Born Surface Area (MM-GBSA) calculations were performed using the gmx_MMPBSA tool (version 1.6.3), which is based on the MMPBSA.py module of AmberTools. Binding free energy ($\Delta G_{\mathrm{bind}}$) was evaluated over 18 structural frames extracted equidistant from the production trajectory.

The total binding free energy was calculated according to the following equation:

$$\Delta G_{\mathrm{bind}}=\left\langle G_{\mathrm{complex}}\right\rangle \;-\;\left\langle G_{\mathrm{receptor}}\right\rangle \;-\;\left\langle G_{\mathrm{ligand}}\right\rangle$$

$$\Delta G_{bind}=\Delta G_{gas}+\Delta G_{solv}$$

$$\Delta G_{gas}=\Delta E_{elec}+\Delta E_{vdW}$$

$$\Delta G_{solv}=\Delta G_{GB}+\Delta G_{surf}$$

where each free energy term encompasses the gas-phase molecular mechanics energy ($\Delta G_{\mathrm{gas}}$, comprising electrostatic ($\Delta E_{elec}$) and van der Waals ($\Delta E_{vdW}$) interactions) and the solvation free energy ($\Delta G_{\mathrm{solv}}$). The solvation component was further partitioned into polar ($\Delta G_{\mathrm{GB}}$) and non-polar ($\Delta G_{\mathrm{surf}}$) solvation energies. The polar solvation contribution was determined using the Generalized Born (GB) implicit solvent model at a simulation temperature of 298.15 K. The non-polar SASA contribution was estimated via the Linear Combination of Pairwise Overlaps (LCPO) empirical surface area algorithm. All calculations were executed utilizing the single-trajectory protocol, assuming no significant conformational rearrangement between the bound and unbound states of the receptor and ligand.

### 2.10. Computational Environment and Statistical Analysis

Custom Python (version 3.10) scripts utilized for data pre-processing and Random Forest model pipeline construction were drafted and optimized with the computational assistance of large language models (i.e., Gemini, Google, Mountain View, CA, USA). All generated code and machine learning architectures were rigorously reviewed, debugged, and validated by the authors prior to execution.

## 3. Results

### 3.1. Transcriptomic Profiling Reveals That VTRNA1-1 Depletion Dismantles Critical Oncogenic and Cell Cycle Networks in AML

**Figure 1:**
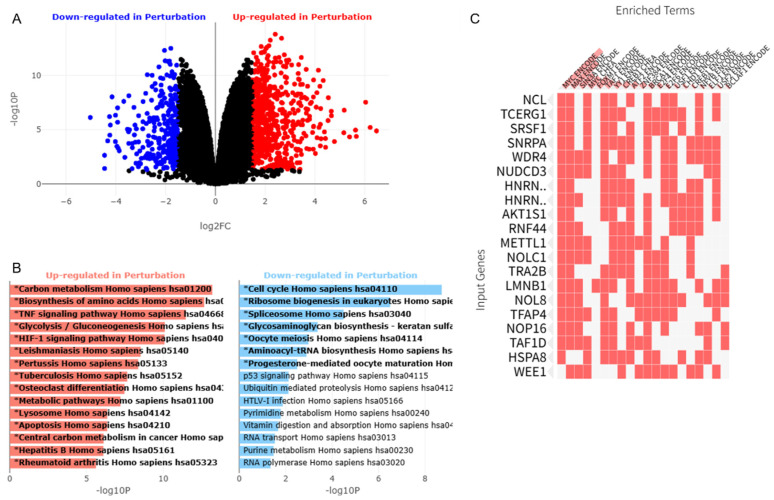
Global transcriptomic profiling reveals that vault RNA1-1 depletion suppresses critical oncogenic networks and the MYC/FOXM1 regulatory axes in acute myeloid leukemia. (**A**) Volcano plot illustrating the differentially expressed genes (DEGs) following vault RNA1-1 (VTRNA1-1) depletion in THP-1 acute myeloid leukemia (AML) cells. Statistically significant upregulated and downregulated transcripts (|log_2_ (Fold Change)| > 1.0, adjusted *p* < 0.05) are highlighted, indicating the broad transcriptional reprogramming induced by VTRNA1-1 loss. (**B**) Functional pathway enrichment analysis using Kyoto Encyclopedia of Genes and Genomes (KEGG) and Gene Ontology of the significantly downregulated DEGs. The bar chart ranks the top disrupted biological processes, showing marked inhibition of mitotic cell cycle progression, DNA replication, and ribosome biogenesis. (**C**) Transcription factor enrichment analysis based on the ENCODE and ChEA consensus databases. The clustergrammer heatmap highlights the top enriched master regulators, demonstrating the systemic collapse of the MYC, MAX, and FOXM1 transcriptional axes upon VTRNA1-1 depletion. * *p* < 0.05 for significant pathways, red: up-regulated, blue: down-regulated.

To establish the biological dependency of AML cells on VTRNA1-1, we generated stable knockdown cell lines utilizing shRNA-expressing lentiviral vectors. The successful depletion of VTRNA1-1 was confirmed via qPCR (Figure S1). To define the global intracellular dependencies and oncogenic signaling networks regulated by VTRNA1-1 in AML, we analyzed high-throughput RNA-seq datasets derived from VTRNA1-1 depletion models (Figure S2). Differential expression analysis comparing VTRNA1-1-depleted THP-1 cells with control cells revealed significant transcriptional reprogramming. Using predefined statistical thresholds (|log_2_ (Fold Change)| > 1.0, adjusted *p*-value < 0.05), we identified a comprehensive signature of DEGs comprising a robust set of significantly upregulated and downregulated transcripts (Figure 1A). To investigate the functional consequences of this transcriptional shift, programmatic pathway enrichment analysis was performed. GO and KEGG pathway analyses demonstrated that downregulated DEGs were significantly enriched in pathways governing mitotic cell cycle progression, DNA replication, ribosome biogenesis, and RNA processing (Figure 1B). Conversely, upregulated transcripts were associated with myeloid differentiation and the induction of apoptosis. To further elucidate the master transcriptional regulators driving the systemic collapse of the leukemic proliferative capacity, we performed transcription factor enrichment analysis using the ENCODE and ChEA consensus databases. Significantly, this analysis revealed significant enrichment of downregulated gene signatures controlled by the MYC, MAX, and FOXM1 transcriptional axes (Figure 1C). Both MYC and FOXM1 are key oncogenic regulators required for leukemic cell proliferation and survival. However, they remain largely “undruggable” through conventional direct inhibition. Collectively, these comprehensive transcriptomic data established that VTRNA1-1 acts as a critical upstream regulator of AML cell survival and suggested that computationally identifying compounds capable of phenocopying this specific DEG signature (the exact input gene lists are detailed in Supplementary Excel File S1) could provide a novel therapeutic strategy to indirectly dismantle the MYC/FOXM1 axis.

### 3.2. Machine Learning and XAI Identify Niclosamide as a Transcriptomic Mimic

**Figure 2:**
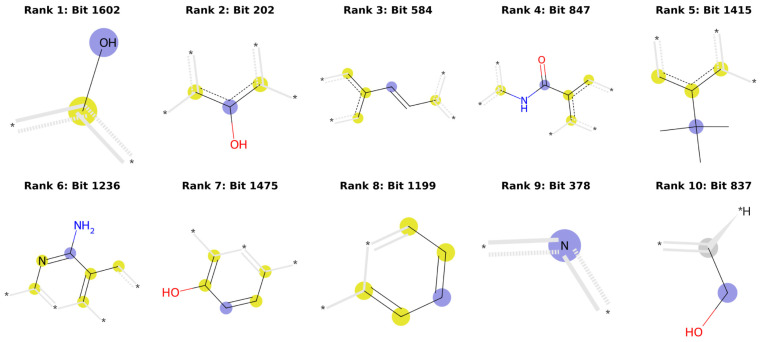
Explainable AI (XAI) analysis revealing the structural determinants of VTRNA1-1 mimics. The top 10 most influential chemical substructures (Morgan fingerprint bits) identified using the trained Random Forest model. These 2D structural fragments represent the critical pharmacophores that the machine learning algorithm relied on to classify compounds as vault RNA1-1 transcriptomic mimics, providing a clear structural rationale for the high ranking of niclosamide and particularly highlighting specific aromatic and amide fragments enriched within its salicylanilide core. Color coding for interactions: green dashed lines, hydrogen bonds; purple dashed lines, π-interactions; red dashed lines, unfavorable contacts; light green circles, van der Waals interactions; blue halos, solvent accessibility. * indicating wildcard attachment points in the chemical substructures.

Having defined the transcriptomic signature of VTRNA1-1 depletion (characterized by MYC/FOXM1 suppression), we hypothesized that the pharmacological induction of this state could offer a novel therapeutic strategy. We first queried the L1000FWD database to establish a dataset of 1461 compounds scored based on their ability to mimic the VTRNA1-1 depletion signature. We translated this dataset into a machine learning pipeline to expand our search across all approved drugs. Using RDKit to convert molecular structures (SMILES) into 2048 bit Morgan fingerprints, we trained a Random Forest classifier. The model achieved a robust predictive accuracy of 71.67% (precision, 0.69; recall, 0.69) for an unseen test set. We subsequently deployed this model to virtually screen the ChEMBL database for existing drugs in phase IV. This AI-driven screening successfully ranked an anthelmintic drug, niclosamide, as the third most probable VTRNA1-1 mimic (Table 1). Although not the top-ranked molecules mathematically, it is notable that other top-ranked candidates (amsacrine, etoposide, topotecan, and irinotecan) are well-established cytotoxic topoisomerase inhibitors that are widely utilized in oncology. In contrast, niclosamide was prioritized in downstream structural and biophysical modeling because of its high clinical translatability (an FDA-approved anthelmintic drug possessing a distinct salicylanilide scaffold) and previous studies highlighting its potent but mechanistically ambiguous antileukemic properties. Furthermore, our XAI analysis elucidated the structural rationale behind this prediction, identifying specific 2D substructures within niclosamide that strongly contributed to the “Mimic” classification by the algorithm (Figure 2).

### 3.3. Molecular Docking Suggests Niclosamide as a High-Affinity Ligand for the p62 ZZ Domain

VTRNA1-1 interacts with the scaffold protein p62 (SQSTM1). We hypothesized that niclosamide induces its phenotype by directly targeting this protein, specifically its structurally critical ZZ domain (PDB ID: 4MJM). To test this hypothesis, blind molecular docking simulations were performed using PyRx (version 0.8). The simulation revealed that niclosamide docks onto the p62 ZZ domain with a thermodynamically favorable docking score of −7.5 kcal/mol (Table 2), which aligns with the conventional benchmark for stable target–ligand configurations.

**Figure 3:**
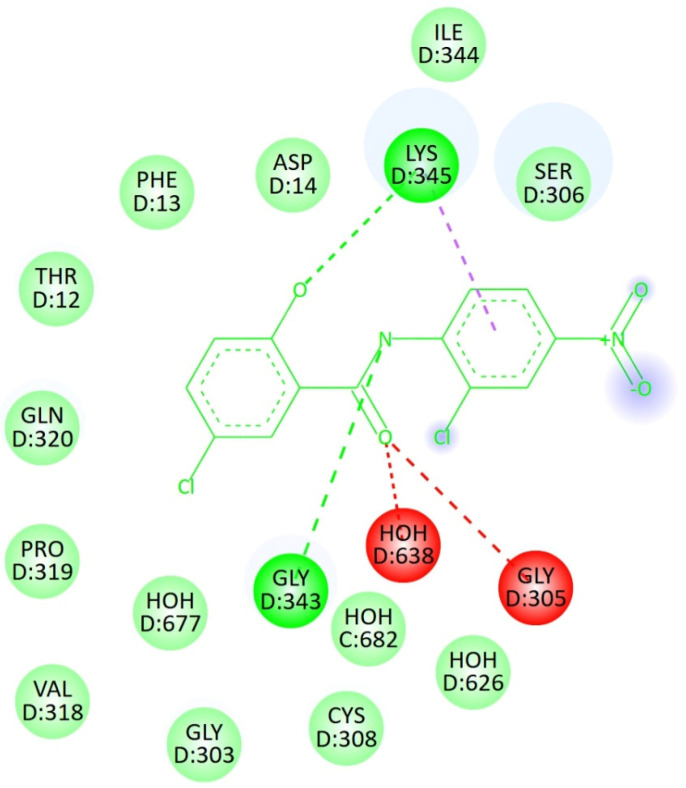
Two-dimensional interaction map of niclosamide docked into the active pocket of the p62 ZZ domain. Visualization of the optimal docking pose (Mode 1) generated using BIOVIA Discovery Studio. The diagram illustrates the specific intermolecular forces stabilizing the complex. Notably, niclosamide forms a strong conventional hydrogen bond (green dashed line) with GLY 343 and a pi interaction (purple dashed line) with LYS 345. Surrounding native amino acid residues, including CYS 308, further anchor the compound within the functional pocket via van der Waals interactions (light green circles). Residues ASP 14 and PHE 13 appear in the contact map as artifacts resulting from crystal packing in PDB 4MJM, and do not represent functional interactions within the core binding pocket of the p62 ZZ domain.

In-depth structural visualization using BIOVIA Discovery Studio was performed to characterize the precise molecular interactions that drive this binding propensity (Figure 3). Niclosamide occupies the active pocket of the ZZ domain. Crucially, its amide group forms a strong conventional hydrogen bond with GLY 343, firmly anchoring the molecule. Simultaneously, the aromatic rings of niclosamide were stabilized through pi–sigma interactions with LYS 345. Supplementary stabilization within the pocket is driven by a dense network of van der Waals forces from native residues, including CYS 308. These initial structural findings suggested that niclosamide possesses the geometric and chemical complementarity required to act as a physical steric barrier over the p62 ZZ domain, potentially blocking its native interaction surfaces and inducing the subsequent phenotypic collapse of downstream pro-survival signaling.

### 3.4. Molecular Dynamics (MD) Simulations Suggest Dynamic Stability and Secondary Structure Integrity of the Complex

**Figure 4:**
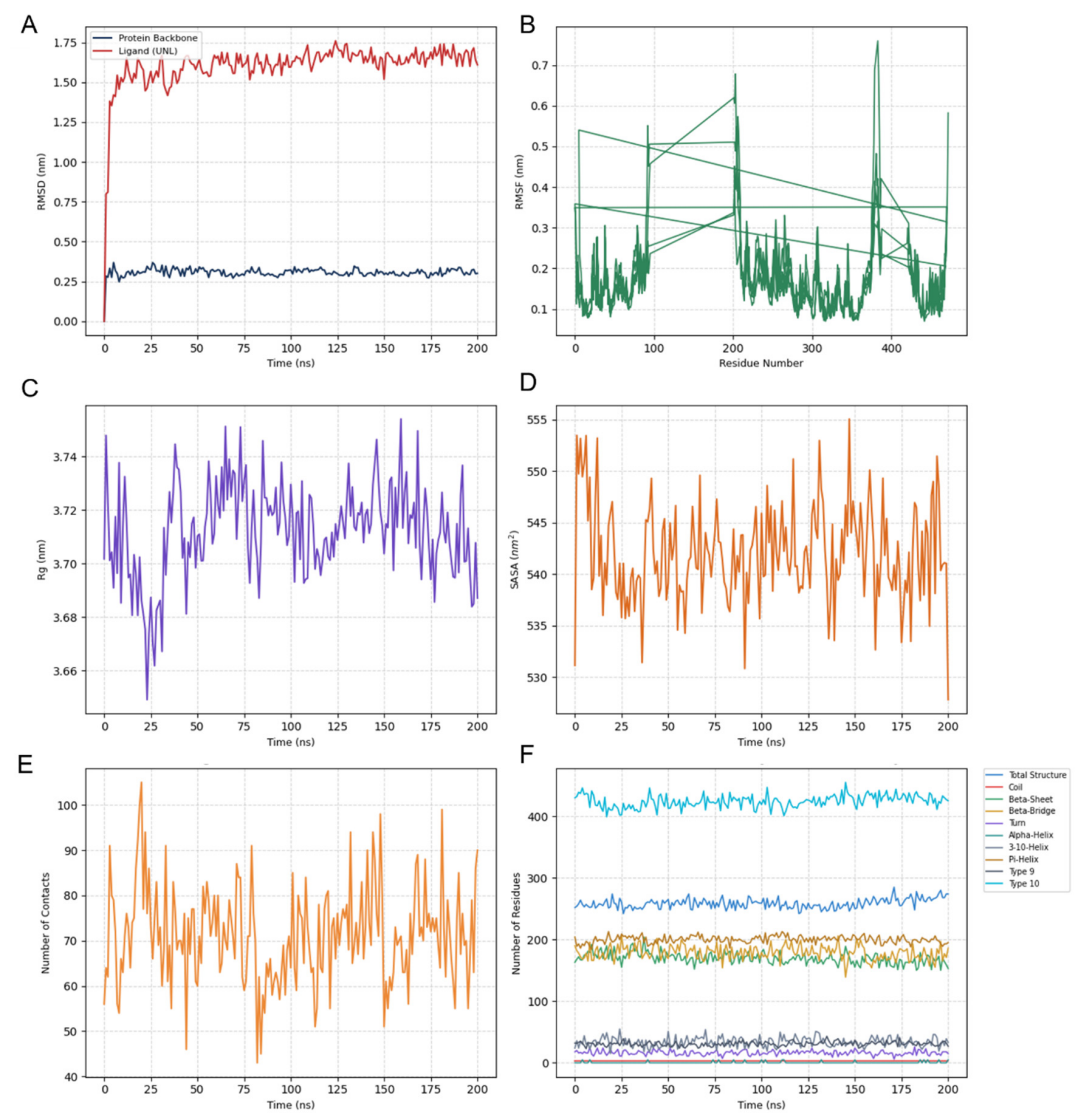
Comprehensive 200 ns molecular dynamics (MD) simulation trajectories characterizing the dynamic stability and structural integrity of the p62 ZZ domain–niclosamide complex. (**A**) Root-mean-square deviation (RMSD) profiles of the protein backbone (navy) and the ligand niclosamide (red), showing rapid equilibration and dynamic convergence. (**B**) Root-mean-square fluctuation (RMSF) profile across the protein sequence; gaps at residues 95–200 and 390–420 indicate non-resolved, intrinsically disordered regions omitted to ensure data integrity, while pocket-forming residues demonstrate minimal flexibility (<0.20 nm). (**C**) Radius of Gyration (Rg) and (**D**) solvent-accessible surface area (SASA) profiles as a function of time, reflecting the strict preservation of the tightly folded, native globular conformation and shielded hydrophobic core. (**E**) Total number of close atomic contacts within a 0.35 nm threshold, illustrating a dense, uninterrupted anchoring network averaging ~73 contacts. (**F**) Time-dependent secondary structure evolution tracked using the gmx dssp module, confirming that ligand binding does not induce local unfolding or structural destabilization.

To evaluate the structural durability and dynamic behavior of the p62 ZZ domain upon niclosamide binding under simulated physiological conditions, a 200 ns all-atom MD simulation was performed using GROMACS 2026. The protein–ligand complex reached structural equilibrium rapidly, as demonstrated by the root-mean-square deviation (RMSD) profiles (Figure 4A). The protein backbone RMSD exhibited a rapid initial rise during the first 10 ns, subsequently stabilizing and achieving a well-converged plateau at approximately 0.30 nm for the remainder of the simulation. This steady profile indicated that the receptor maintained a highly stable and rigid global conformation without undergoing detrimental structural denaturation. The ligand RMSD reached approximately 1.65 nm after 25 ns and subsequently remained stable without sustained drift or sudden fluctuations between 25 ns and 200 ns, suggesting that the ligand did not dissociate from the binding pocket, successfully adapting into a dynamically optimized, highly robust binding conformation. To map the local flexibility of individual amino acid residues, the root-mean-square fluctuation (RMSF) was plotted across the protein sequence (Figure 4B). Notably, distinct gaps in the trajectory profile were observed at residues 95–200 and 390–420, which correspond to the intrinsically disordered loop regions that were unresolved in the original crystal structure of the human p62 ZZ domain (PDB ID: 4MJM) and were intentionally excluded from the analysis to prevent structural artifacts. Within the experimentally determined core regions, the residues constituting the niclosamide-binding pocket exhibited low fluctuations (consistently remaining below 0.20 nm), underscoring the stabilizing effect induced by the strong intermolecular interaction network. In contrast, localized peaks exceeding 0.40 nm were confined exclusively to highly solvent-exposed loops adjacent to the missing segments and the flexible terminal residues. The structural compactness and solvent exposure of the complex were evaluated using the Radius of Gyration (Rg) and solvent-accessible surface area (SASA). The Rg profile remained stable throughout the 200 ns timeline, fluctuating within a narrow range of 3.68 nm to 3.74 nm (Figure 4C). This steady trend confirms that the protein did not undergo unfolding or significant conformational swelling. Consistent with the Rg data, the total SASA values remained stable and equilibrated between 535 and 550 nm^2^ (Figure 4D). The maintenance of a stable surface area indicates that the hydrophobic core and the internal binding pocket enclosing niclosamide remained securely shielded from the bulk solvent. The longevity and persistence of the binding mode were evaluated by quantifying the number of close atomic contacts (within a 0.35 nm threshold) as a proxy for hydrogen bond occupancy and steric fit (Figure 4E). The contact frequency remained high and uninterrupted throughout the 200 ns timescale, maintaining an average of approximately 73 close contacts without dropping to zero, confirming continuous binding. The transient decrease observed at approximately 80 ns corresponds to the local orientational optimization of the ligand inside the pocket, which was promptly restabilized. Finally, the impact of niclosamide binding on local folding of the receptor was analyzed using time-dependent secondary structure tracking using the integrated gmx dssp module (Figure 4F). The residue counts for the primary secondary structural elements, including α-helices, β-sheets, and turns, remained essentially unchanged throughout the simulation, indicating that niclosamide binding does not induce conformational destabilization or local unfolding of the host protein, establishing that niclosamide may function as a persistent physical “lid” over the p62 ZZ domain cleft.

### 3.5. Binding Free Energy Analysis via MM-GBSA

**Figure 5:**
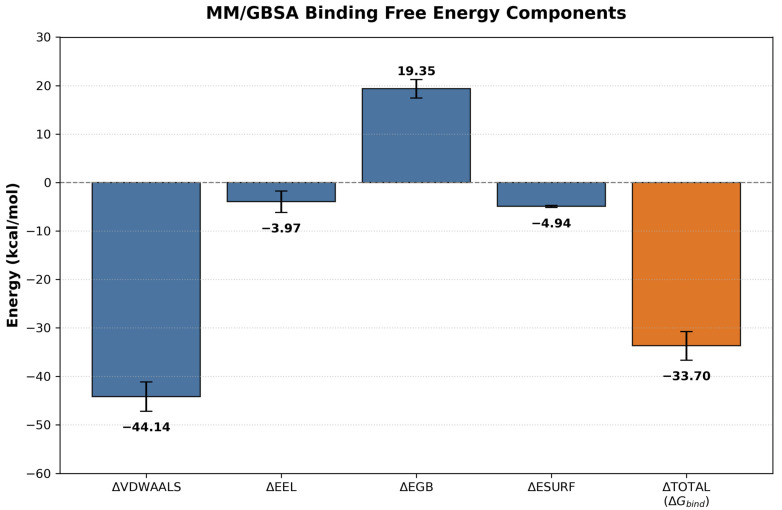
MM-GBSA binding free energy decomposition for the p62 ZZ domain–niclosamide complex. The individual thermodynamic components and the net binding free energy (ΔTOTAL or $\Delta G_{\mathrm{bind}}$) evaluated over 18 equidistant structural frames extracted from the 200 ns MD production trajectory are presented in kcal/mol. Each bar represents the ensemble average of a specific energetic contribution: van der Waals interactions (ΔVDWAALS), electrostatic interactions (ΔEEL), polar solvation free energy (ΔEGB), non-polar surface solvation free energy (ΔESURF), and the total estimated binding affinity (ΔTOTAL). Error bars denote the sample standard deviation (SD) across the evaluated trajectory frames. The pronounced negative contributions of ΔVDWAALS and ΔESURF highlight a robust hydrophobic-driven binding mechanism, which effectively overcomes the unfavorable polar desolvation penalty (ΔEGB) to yield a highly exergonic and stable overall complexation.

To quantitatively corroborate the thermodynamic stability and evaluate the driving forces governing the molecular recognition between the p62 ZZ domain and niclosamide, endpoint free energy calculations were implemented using the MM-GBSA approach. The total binding free energy ($\Delta G_{\mathrm{bind}}$) and its individual energetic components averaged over the selected production trajectory frames are summarized in Table 3. The overall binding free energy was determined to be − 33.70±2.92 kcal/mol (Figure 5), demonstrating an exceptionally strong, spontaneous, and thermodynamically favorable binding affinity under simulated physiological conditions. This robust negative value serves as an energetic validation for the structural persistence and high atomic contact frequency (average of ~73 close contacts) documented in the preceding structural trajectory analysis.

Decomposition of the binding free energy into specific physical components revealed that the interaction is predominantly driven by non-polar van der Waals forces. The van der Waals contribution ($\Delta E_{\mathrm{vdw}}$) exerted a powerful stabilizing effect of − 44.14±3.03 kcal/mol, representing the primary thermodynamic anchor for the complex. This pronounced negative contribution strongly indicates that the aromatic rings and hydrophobic substituents of the niclosamide scaffold establish an optimized, shape-complementary network of hydrophobic interactions and steric packing within the hydrophobic cleft of the p62 ZZ domain structure. The non-polar solvation energy ($\Delta E_{\mathrm{surf}}$), estimated at − 4.94±0.21 kcal/mol, further reinforced this hydrophobic-driven binding mode, reflecting the favorable classical hydrophobic effect elicited by the burial of solvent-accessible surface areas upon complexation, consistent with the steady SASA profiles.

In contrast, the electrostatic components displayed a delicate thermodynamic trade-off between gas-phase Coulombic attractions and solution-phase desolvation costs. The intermolecular gas-phase electrostatic interaction ($\Delta E_{\mathrm{el}}$) provided a modest favorable contribution of − 3.97±2.21 kcal/mol. However, this electrostatic gain was heavily countered by a substantial polar solvation energy penalty ($\Delta E_{\mathrm{GB}}$) of +19.35±1.92 kcal/mol. This large unfavorable positive value represents the inevitable desolvation penalty incurred by stripping the polar water shell away from the highly solvent-exposed binding cleft of the p62 ZZ domain and the functional groups of niclosamide during the docking event. Crucially, because the magnitude of the desolvation penalty (+19.35 kcal/mol) is vastly outweighed by the overwhelming stabilization derived from the hydrophobic terms ($\Delta E_{\mathrm{vdw}}+\Delta E_{\mathrm{surf}}\approx \;-\;49.08\;\mathrm{kcal}/\mathrm{mol}$), the complex yields a highly exergonic total net binding energy. Collectively, these thermodynamic profiles unequivocally characterize the p62 ZZ domain–niclosamide association as a classic hydrophobic-driven binding process, substantiating the role of niclosamide as a highly stable, physical “lid” over the functional binding domain.

## 4. Discussion

AML remains an aggressive hematological malignancy, largely due to the therapeutic resistance and the persistent activity of undruggable oncogenic drivers such as MYC [24,25,26,27,28,29,30,31]. In this study, we identified the non-coding RNA VTRNA1-1 as a potential therapeutic vulnerability in AML. Our integrated computational framework and transcriptomic profiling demonstrated that VTRNA1-1 depletion was associated with impaired proliferative programs in AML cells. Notably, RNA-seq and subsequent transcriptomic network analyses revealed that VTRNA1-1 depletion was associated with the broad disruption of the MYC and FOXM1 signaling axes. Because directly targeting transcription factors like MYC has historically been exceptionally challenging, inducing a “VTRNA1-1-depleted state” via pharmacological intervention presents a highly attractive, alternative therapeutic hypothesis.

To translate this transcriptomic vulnerability into a potential therapeutic strategy, we developed a machine-learning-based virtual screening pipeline. A Random Forest model trained on the L1000FWD transcriptomic database was used to evaluate approved drugs for their ability to mimic the transcriptional effects of VTRNA1-1 depletion. Notably, several established topoisomerase inhibitors, including amsacrine and etoposide, ranked among the top candidates. Although this computationally validated that our model correctly identified compounds capable of inducing severe cell stress and death in leukemic blasts, these traditional chemotherapeutics are notorious for their broad systemic toxicity and secondary malignancies. Crucially, in L1000FWD transcriptomic datasets, cytotoxic agents often cluster together simply because they induce broad cell stress responses. To actively mitigate this generic cytotoxicity bias and isolate true VTRNA1-1/p62-driven transcriptomic phenocopying from non-specific stress signatures, we strategically prioritized the third-ranked candidate, niclosamide. Niclosamide, an FDA-approved anthelmintic drug, has a well-established safety profile that makes it an ideal candidate for drug repositioning. Furthermore, XAI analysis successfully supported this prediction by identifying specific 2D chemical pharmacophores (e.g., its salicylanilide core) that mimic the biological signature of VTRNA1-1 depletion, thereby validating the structural rationale behind the algorithm’s choice.

Although niclosamide has been reported to exhibit antitumor properties in a variety of cancers, its molecular mechanism of action in AML remains incompletely understood [32,33,34,35,36]. Given the reported interaction between VTRNA1-1 and the autophagic receptor p62 (SQSTM1), we hypothesized that niclosamide may modulate this regulatory axis. Blind molecular docking identified a favorable binding pose for niclosamide within the active functional pocket of the p62 ZZ domain, with a predicted binding affinity of −7.5 kcal/mol. The formation of a stable conventional hydrogen bond with GLY 343, reinforced by pi–sigma interactions with LYS 345, positions niclosamide as a structural “lid” over the ZZ domain framework. We propose that, by sterically occluding this domain, niclosamide prevents p62 from interacting with essential client proteins. This physical blockade likely disrupts downstream pro-survival cascades, ultimately culminating in the transcriptomic collapse of the MYC and FOXM1 networks, as observed in our RNA-seq data.

To bridge the gap between static in silico docking and the thermodynamic realities of simulated physiological fluids, a 200 ns MD simulation was executed. The simulation demonstrated that niclosamide functions not merely as a temporary interactor, but as a persistent structural “lid” capable of sustained steric occlusion within the p62 ZZ domain. On a global scale, this complex achieves rapid energy and conformational convergence. The protein backbone RMSD settled at ~0.30 nm, while the highly stable Radius of Gyration (Rg: 3.68–3.74 nm) and solvent-accessible surface area (SASA: 535–550 nm^2^) baselines confirmed that the p62 ZZ domain preserved its compact, native globular fold without undergoing unfavorable unfolding or structural expansion. Microscopically, the local flexibility was strictly suppressed (< 0.20 nm) within the functional core. Notably, the distinct gaps observed in the RMSF plot (residues 95–200 and 390–420) aligned perfectly with the intrinsically disordered regions or missing flexible loops characteristic of full-length p62, which are frequently unresolved in crystallography (PDB ID: 4MJM) due to their inherent structural plasticity. Intentional exclusion of these unresolved segments ensured that the dynamic readings strictly represented the experimentally verified binding core of the domain. Biochemically, the exceptional longevity of this complex was driven by a high-density interaction network, maintaining an average of 73 close atomic contacts (within 0.35 nm) that never dropped to zero. While our static docking identified initial hydrogen bonding with GLY 343 and pi–sigma interactions with LYS 345, the 200 ns MD trajectory revealed a significant dynamic adaptation, wherein the ligand’s hydrophobic salicylanilide core underwent rigid-body reorientation, settling into a strictly flat plateau at an RMSD of ~1.65 nm. In biomolecular simulations, such an elevated yet perfectly horizontal ligand baseline signifies a transition from an initial metastable docking coordinate to a deeper, thermodynamically optimized energy minimum. This thermodynamic stability was further supported by our MM-GBSA endpoint free energy calculations, which yielded an exceptionally favorable net binding affinity ($\Delta G_{\mathrm{bind}}$) of −33.70±2.92 kcal/mol. Energy decomposition confirmed that this robust complexation is overwhelmingly driven by van der Waals interactions (ΔVDWAALS= −44.14±3.03 kcal/mol) and non-polar surface burial (ΔESURF= −4.94±0.21 kcal/mol), providing quantitative support that the hydrophobic salicylanilide core of niclosamide achieves tight, shape-complementary packing within the target cleft. This structural and thermodynamic transition allows niclosamide to slide into the deep acidic cavity of the ZZ domain, which is evolutionarily conserved and recognizes type-1 and type-2 N-degrons (e.g., N-terminal arginine) [37,38]. This dynamic occlusion of the N-degron-binding surface provides a highly compelling molecular rationale for the downstream transcriptome effects observed in our study. Although the prior literature indicates that VTRNA1-1 physically binds to the N-terminal PB1 domain of p62 (specifically at Lys7 and Arg21) to regulate oligomerization [21], our machine learning L1000FWD pipeline was strategically designed to screen for compounds that phenocopy the global transcriptomic signature of VTRNA1-1 depletion rather than simple competitive binders. By chemically sealing the highly conserved ZZ domain cleft, niclosamide structurally locks p62, preventing it from engaging with its essential N-degron client proteins and crosstalk with the autophagic machinery [39]. This blockade may significantly disrupt the p62-mediated pro-survival signaling hubs. This functional collapse underpins the systemic shutdown of the downstream MYC and FOXM1 networks observed in our RNA-seq data, providing a robust, multidisciplinary rationale for niclosamide as a targeted therapeutic agent in AML.

Despite these promising findings, our study had certain limitations that should be acknowledged. First, the transcriptomic signature was derived from a single AML cell line model (THP-1) with a specific genetic modification; thus, independent functional validation across diverse AML molecular subtypes is essential in future studies. Second, definitive biophysical confirmation of p62–niclosamide interactions, such as Surface Plasmon Resonance (SPR) and p62 mutant rescue experiments, and rigorous quantification of binding free energy remain necessary. Furthermore, the current evidence is primarily derived from advanced in silico models and retrospective public datasets. The BM microenvironment plays a pivotal role in AML progression and drug resistance, yet its complexity cannot be fully recapitulated by computational models or conventional 2D cell culture systems. Therefore, future in vivo studies utilizing patient-derived xenograft (PDX) murine models are needed to evaluate the pharmacokinetic profile, optimal dosing, and physiological efficacy of niclosamide as a VTRNA1-1 mimic.

## 5. Conclusions

In conclusion, this data-driven study successfully integrated transcriptomic profiling, AI-driven drug repositioning, and structural biology to propose a potential vulnerability in AML. We provided a comprehensive computational and biological rationale for repurposing niclosamide, suggesting that it is a potential p62 ZZ domain binder capable of phenocopying the VTRNA1-1 depletion state. These findings not only provide preliminary insights into the VTRNA1-1/p62 regulatory axis but also highlight the potential of AI-driven approaches to accelerate the discovery and prioritization of novel leukemia therapeutics, providing a hypothesis-generating foundation that warrants rigorous experimental validation.

## Figures and Tables

**Table 1:** Top 5 candidate compounds identified by the Random-Forest-based virtual screening mimicking the VTRNA1-1 knockdown transcriptomic signature.

Rank	Drug Name (ChEMBL-Approved)	Mimic Probability (%)
1	AMSACRINE	99.25
2	ETOPOSIDE	99.00
3	NICLOSAMIDE	98.17
4	TOPOTECAN	96.00
5	IRINOTECAN	95.00

**Table 2:** Top three binding poses and affinities of niclosamide docked into the human p62 (SQSTM1) ZZ domain (PDB ID: 4MJM).

Mode	Binding Affinity (kcal/mol)	RMSD (Lower Bound)	RMSD (Upper Bound)
1	−7.5	0	0
2	−7.5	3.106	7.679
3	−7.3	2.232	2.515

**Table 3:** Binding free energy components for the complex.

Component	Average	SD	Unit
ΔVDWAALS	−44.14	3.03	kcal/mol
ΔEEL	−3.97	2.21	kcal/mol
ΔEGB	19.35	1.92	kcal/mol
ΔESURF	−4.94	0.21	kcal/mol
ΔTOTAL (ΔG_bind)	−33.70	2.92	kcal/mol

ΔVDWAALS: van der Waals interaction energy, ΔEEL: electrostatic interaction energy, ΔEGB: polar solvation free energy determined via the Generalized Born implicit solvent model, ΔESURF: non-polar (surface) solvation free energy estimated using the Linear Combinations of Pairwise Overlaps (LCPO) algorithm, ΔGGAS: total gas-phase molecular mechanics energy (ΔVDWAALS+ΔEEL, ΔGSOLV: total solvation free energy change (ΔEGB+ΔESURF), ΔTOTAL ($\Delta G_{\mathrm{bind}}$): total net binding free energy, SD: standard deviation.

## Data Availability

Data are contained within the article or Supplementary Materials.

## Supplementary Materials

The following supporting information can be downloaded at: https://www.mdpi.com/article/10.3390/biology15151290/s1, Figure S1: Validation of VTRNA1-1 knockdown efficiency in THP-1 cells; Figure S2: Library size analysis of RNA-sequencing samples; Table S1: Nucleotide sequences of shRNAs and qPCR primers used in this study; Supplementary Excel File S1.
