# Supplementary material for: Molecular Docking and Simulation-Based Exploration of Niclosamide as a Potential Inhibitor of the p62 ZZ Domain
Source: Biology (Basel). 2026 Aug 4;15(15):1290. doi: 10.3390/biology15151290 (PMC13465034; doi:10.3390/biology15151290)
Supplement: Supplementary file 1 [file biology-15-01290-s001.zip › Figures S1&S2&Table S1.pdf]

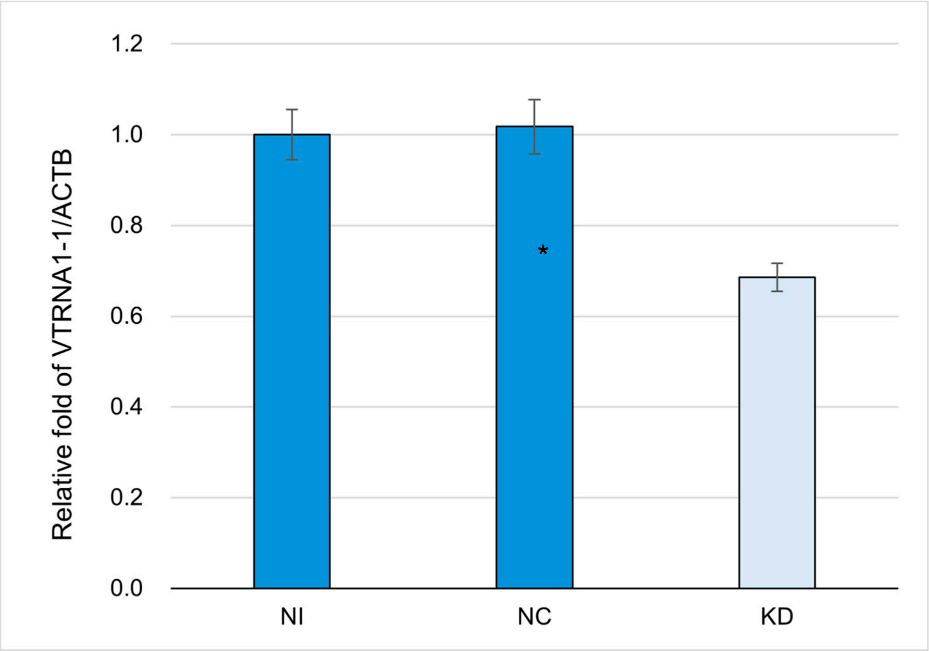

Figure S1. Validation of VTRNA1-1 knockdown efficiency in THP-1 cells. THP-1 cells were transduced with lentiviral vectors expressing either a scrambled negative control shRNA (sh-NC) or a VTRNA1-1-targeting shRNA (sh-VTRNA1-1). The relative expression levels of VTRNA1-1 were evaluated by quantitative real-time PCR (qPCR) and normalized to ACTB (β-actin) as an endogenous control. Data are presented as the mean ± standard deviation (SD) of three independent experiments (n = 3). Statistical significance was determined using Student's t-test (* $p < 0.05$ vs. sh-NC).

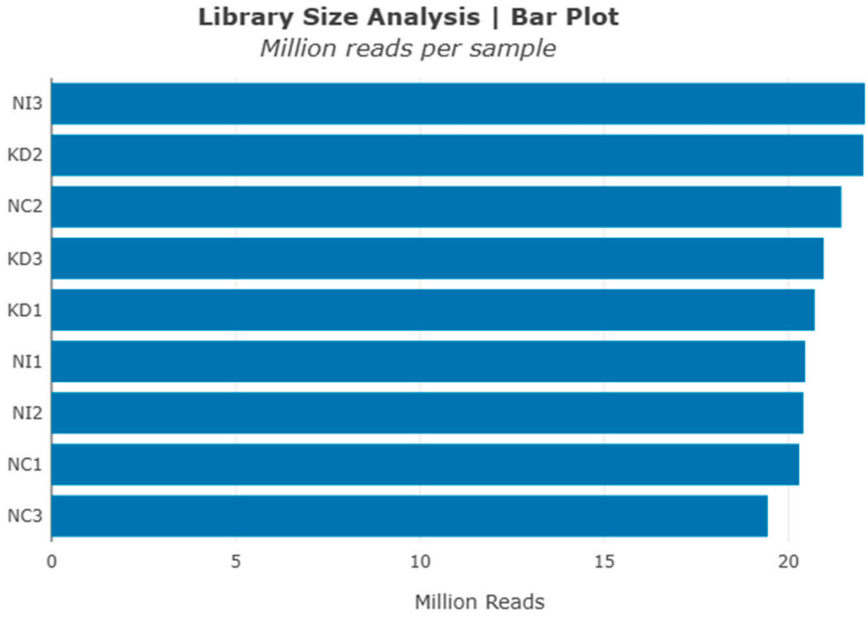

Figure S2. Library size analysis of RNA-sequencing samples. Bar plot illustrating the total number of sequencing reads (in millions) obtained for each individual sample, demonstrating consistent sequencing depth and high-quality library preparation across the control (NC), knockdown (KD), and treated (NI) groups.

Table S1. Nucleotide sequences of shRNAs and qPCR primers used in this study.

Target / Gene	Type	Sequence (5' to 3')
sh-VTRNA1-1	shRNA	ATTACTTCGACAGTTCTTTAATTAGTGAA-GCCACAGATGTAATTAAAGAACTGTCGAAGTAAC
sh-NC	shRNA	CCCTAAGGTTAAGTCGCCCTCGTAGTGAA-GCCACAGATGTACGAGGGCGACTTAACCTTAGGT
VTRNA1-1	Forward Primer	GGCTGGCTTTAGCTCAGCG
VTRNA1-1	Reverse Primer	AAGGACTGGAGAGCGCCC
VTRNA1-1	Probe	^{FAM}CAAGCAACCTGTCTGGGTTGTTCGAGACC^{TAM}
ACTB	Forward Primer	CTCGCCTTTGCCGATCCG
ACTB	Reverse Primer	CATGCCGGAGCCGTTGTC
ACTB	Probe	^{HEX} HEXACACCCGCCGCCAGCTCACCAT ^{TAM}

FAM, fluorescein amidites; HEX, Hexachlorofluorescein; TAM, tetramethylrhodamine.
